# Arabidopsis *SMALL DEFENSE-ASSOCIATED PROTEIN 1* Modulates Pathogen Defense and Tolerance to Oxidative Stress

**DOI:** 10.3389/fpls.2020.00703

**Published:** 2020-06-03

**Authors:** Aditya Dutta, Pratibha Choudhary, Pallavi Gupta-Bouder, Snigdha Chatterjee, Po-Pu Liu, Daniel F. Klessig, Ramesh Raina

**Affiliations:** ^1^Department of Biology, Syracuse University, Syracuse, NY, United States; ^2^Boyce Thompson Institute, Ithaca, NY, United States

**Keywords:** *SMALL DEFENSE-ASSOCIATED PROTEIN 1*, *SDA1*, salicylic acid, bacterial pathogen, defense signaling, *Arabidopsis thaliana*, ROS, abiotic stress

## Abstract

Salicylic acid (SA) and reactive oxygen species (ROS) are known to be key modulators of plant defense. However, mechanisms of molecular signal perception and appropriate physiological responses to SA and ROS during biotic or abiotic stress are poorly understood. Here we report characterization of *SMALL DEFENSE-ASSOCIATED PROTEIN 1* (*SDA1*), which modulates defense against bacterial pathogens and tolerance to oxidative stress. *sda1* mutants are compromised in defense gene expression, SA accumulation, and defense against bacterial pathogens. External application of SA rescues compromised defense in *sda1* mutants. *sda1* mutants are also compromised in tolerance to ROS-generating chemicals. Overexpression of *SDA1* leads to enhanced resistance against bacterial pathogens and tolerance to oxidative stress. These results suggest that SDA1 regulates plant immunity via the SA-mediated defense pathway and tolerance to oxidative stress. *SDA1* encodes a novel small plant-specific protein containing a highly conserved seven amino acid (S/G)WA(D/E)QWD domain at the N-terminus that is critical for SDA1 function in pathogen defense and tolerance to oxidative stress. Taken together, our studies suggest that SDA1 plays a critical role in modulating both biotic and abiotic stresses in Arabidopsis (*Arabidopsis thaliana*) and appears to be a plant-specific stress responsive protein.

## Introduction

Plants frequently encounter pathogenic microbes, yet most of these interactions do not lead to disease. Generally, plants have two levels of defense against pathogens. The first level of defense is activated upon recognition of pathogen-associated molecular pattern (PAMP) that are highly conserved molecules such as flagellin and lipopolysaccharide, commonly present on the surface of several types of bacterial pathogens. Plants upon sensing these PAMPs with receptor kinases on the surface of the plasma membrane initiate PAMP-triggered immunity (PTI), which in turn leads to activation of several downstream defense-associated genes via MAP kinase signaling leading to basal resistance. However, basal defenses are not sufficient to prevent pathogen growth and disease progression. Over time, pathogens have developed mechanisms to suppress PTI by interfering with recognition at the plasma membrane or by secreting effector proteins (also known as virulence factors) directly into the plant cell. In response, plants have evolved resistance (R) proteins, which directly or indirectly detect the presence of effector proteins. This detection results in the induction of a more rapid and robust defense response known as *R* gene-mediated response or effector–triggered immunity (ETI) (Chisholm et al., 2006; Bent and Mackey, 2007; Zipfel, 2009; Dodds and Rathjen, 2010).

A frequent outcome of ETI is activation of the hypersensitive response (HR). HR is a form of programmed cell death (PCD) induced at the site of pathogen infection. HR is usually accompanied by the synthesis of antimicrobial compounds, strengthening of the cell walls, activation of several defense genes, production of reactive oxygen species (ROS), changes in ion fluxes and protein phosphorylation, expression of pathogenesis-related (PR) genes, and the eventual death of the infected cell and the pathogen (Jones and Dangl, 2006; Dodds and Rathjen, 2010; Gust et al., 2012; Boyd et al., 2013; Wirthmueller et al., 2013; Mauch-Mani et al., 2017). In most cases, HR is followed by the activation of a long-lasting, broad-spectrum resistance termed as systemic acquired resistance (SAR), which is characterized by elevated levels of salicylic acid (SA) and expression of PR genes in the systemic tissues. In plants, SA is produced from chorismate primarily by two known biosynthetic pathways. In the first case, chorismate is converted to SA via phenylalanine, whereas in the second case, chorismate is converted to SA via isochorismate. In Arabidopsis (*Arabidopsis thaliana*), tobacco (*Nicotiana tabacum*), and tomato (*Solanum lycopersicum*) the bulk of the pathogen-induced SA is synthesized via chorismate. In plants, SA is likely to be produced in chloroplasts and the bulk of it is stored in vacuoles as inactive SA O-β-glucoside (SAG), which can be converted back to active SA (Wildermuth, 2006; Chen et al., 2009; Vlot et al., 2009). Additionally, SA is also present as a conjugate with amino acids (Staswick et al., 2002, 2005; Okrent et al., 2009).

SA and its derivatives play a critical role in modulating local and systemic resistance against biotrophic and hemi-biotrophic microbial pathogens. Plants that are defective in biosynthesis and accumulation of SA or signaling mediated by SA are compromised in resistance against pathogens (Durrant and Dong, 2004; Vlot et al., 2009). For example, transgenic Arabidopsis and tobacco plants expressing a bacterial salicylate hydroxylase gene (NahG) are unable to accumulate SA and develop SAR, and therefore display enhanced susceptibility to virulent pathogens (Gaffney et al., 1993; Delaney et al., 1994). SA has also been shown to be a key modulator of PTI (Bigeard et al., 2015), given that disruption of SA-mediated signaling affects bacterial flagellin-mediated responses. Arabidopsis mutants such as *eds1*, *pad4*, *sid2*, *sid1*/*eds5*, *gdg1*/*pbs3*/*win3*, and *eps1* are compromised in their ability to accumulate SA in response to pathogen infection and display enhanced susceptibility to pathogens (Glazebrook et al., 1996, 1997; Parker et al., 1996; Rogers and Ausubel, 1997; Nawrath and Metraux, 1999; Dewdney et al., 2000; Feys et al., 2001; Wildermuth et al., 2001; Nawrath et al., 2002; Jagadeeswaran et al., 2007; Lee et al., 2007; Nobuta et al., 2007; Zheng et al., 2009). Additionally, Arabidopsis *npr1/nim1/sai1* mutants are defective in transmitting SA signals, and are also unable to develop SAR and display enhanced susceptibility to virulent pathogens (Cao et al., 1994; Delaney et al., 1995; Shah et al., 1997).

Here, we describe identification and characterization of the *SMALL DEFENSE-ASSOCIATED PROTEIN 1* (*SDA1*) gene of *Arabidopsis thaliana* (hereafter Arabidopsis). We show that *sda1* mutants are compromised in activation of *Pathogenesis-Related* (*PR*) genes, accumulation of SA, resistance against bacterial pathogen *Pseudomonas syringae* (*P. syringae*), and in tolerance against ROS-inducing agents. The *SDA1* gene is predicted to encode a small 86 amino acid long plant-specific protein, which contains a highly conserved seven amino acid (S/G)WA(D/E)QWD domain at the N-terminus. Site-directed mutagenesis revealed the requirement of this domain in modulating bacterial defense and tolerance against oxidative stress in Arabidopsis. Our results show that SDA1 defines a novel class of small plant-specific proteins involved in modulating pathogen defense responses and oxidative stress tolerance.

## Materials and Methods

### Plant Growth, Chemical Treatment, and Pathogen Infection of Plants

Growth of bacterial pathogens, plant infections, and in planta pathogen growth assays were performed as described previously (Dutta et al., 2017). Plants were grown and treated with salicylic acid (SA) as described previously (Jagadeeswaran et al., 2007). For pathogen growth assay, bacterial pathogens were grown at 28°C on LB agar plates or in liquid medium supplemented with 100 μg/mL rifampicin and 50 μg/mL kanamycin. Bacterial cultures were prepared by resuspending the overnight grown cells in 10 mM MgCl_2_ to a titer of 5 × 10^5^ cfu/mL (OD_600_; 1 OD_600_ = 10^9^ cfu/mL). The bacterial suspension was pressure-infiltrated on the abaxial side of the leaves using a 1-mL syringe. Alternatively, whole rosettes were dipped into the bacterial suspension containing 0.02% Silwet L-77 (surfactant) and swirled for 30 s to evenly coat the leaves. For each genotype, eight plants were analyzed individually. Three leaf discs (0.5 cm in diameter) from each plant were collected at indicated times and placed in 1 mL of 10 mM MgCl_2_ in a microfuge tube. Tubes were shaken at 200 rpm for 1 h. Serial dilutions were plated on LB agar plates supplemented with appropriate antibiotics. Plates were incubated at 28°C for 2 days to determine the number of colony-forming units.

### Analysis of *sda1* Insertional Mutants

T-DNA insertion lines *sda1-1* (SAIL_390_F08) and *sda1-2* (GABI_283D01) were obtained from the Syngenta T-DNA collection (ABRC) (Alonso et al., 2003) and GABI-Kat collection (NASC), respectively. Genomic DNA flanking the T-DNA insertion site in *sda1-1* was amplified using SDA1 gene-specific forward primer and T-DNA reverse primer as listed in Supplementary Table S1. Genomic DNA flanking the T-DNA insertion in *sda1-2* was amplified using SDA1 gene specific forward primer and T-DNA reverse primer as listed in Supplementary Table S1. Amplified PCR products were sequenced to determine the site of insertion of the T-DNA. Homozygous null mutants were identified by northern blot analysis. To test the ability of SA to restore resistance to *Pst* DC3000 and *Psm* ES4326 in *sda1* mutants, *sda1* mutants and the corresponding Col-0 were sprayed with water or 1 mM SA 24 h prior to treatment with 5 × 10^5^ cfu/mL *Pst* DC3000 and *Psm* ES4326 suspension prepared in 10mM MgSO_4_ solution, and growth of the pathogen was determined 3 days post-inoculation (dpi), and disease development was monitored for 5 dpi.

### Construction and Analysis of *SDA1* Overexpression and β-Estradiol Inducible-*SDA1* Overexpression Plants

Full length SDA1 cDNA was amplified using Platinum Taq High Fidelity DNA Polymerase (Invitrogen, United States), with forward and reverse primers as listed in Supplementary Table S1, using U11998 SSP pUNI clone (ABRC) as template, and was cloned into pCR8/GW/TOPO TA cloning vector (Invitrogen, United States). Sequence of the amplified product was compared with the mRNA sequences available in the public databases to confirm that correct full-length cDNA was amplified. SDA1 cDNA was cloned in the gateway system pMDC32 vector (ABRC) (Curtis and Grossniklaus, 2003) under control of a strong 35S promoter to engineer SDA1 overexpression mutants. β-estradiol dose-dependent inducible overexpression of SDA1 was engineered by cloning of SDA1 cDNA in the gateway vector pMDC7 (Curtis and Grossniklaus, 2003). All of these constructs were then introduced into wildtype Col-0 plants via Agrobacterium-mediated floral dip transformation method (Clough and Bent, 1998). Transgenic plants were selected on solid Peters media or half-strength MS media with 1% sucrose and agar supplemented with 40 μg/mL hygromycin.

β-estradiol (Sigma, United States) treatment at indicated concentrations in 0.1% ethanol for inducible overexpression of SDA1, were syringe infiltrated into the rosette leaves of 4-week-old plants and tissue samples were harvested for RNA isolation at indicated times.

### Construction and Analysis of *SDA1* Promoter::GUS Transgenic Plants

The SDA1 promoter was isolated as a 2 kb 5’-region upstream of the translation start site of SDA1 by PCR amplification of the genomic DNA using forward and reverse primers as listed in Supplementary Table S1. The amplified product was cloned into pCR8/GW/TOPO TA cloning vector (Invitrogen, United States), and sequenced to confirm the sequence of the amplified product. The promoter was cloned upstream of the GUS gene in the gateway system pMDC163 vector (ABRC) to create a transcriptional fusion SDA1 promoter::GUS. This construct was introduced into Col-0 plants via Agrobacterium-mediated transformation (Clough and Bent, 1998). Transgenic plants were selected on solid Peters media supplemented with 40 μg/mL hygromycin. Histochemical analyses for GUS activity were carried out in 6–8 independent transgenic lines for each tissue. Tissue samples were incubated overnight at 37°C in GUS assay buffer (10 mM phosphate buffer, pH 7; 0.5% Triton X-100; 2 mM potassium ferricyanide; 1 mg/mL X-Gluc) and were cleared in 70% ethanol for 2–3 days.

### Site-Directed Mutagenesis

SDA1 cDNA-pCR8/GW/TOPO TA construct was used as a template to engineer four SDA1-mutant constructs (D8A, Q9R, V27A, and K52A) by using a site-directed mutagenesis kit (Quickchange Kit, Stratagene). Point mutations were confirmed by DNA-sequencing using oligonucleotide primers listed in Supplementary Table S1. These four mutants were further sub-cloned into pMDC32 (under cauliflower mosaic virus 35S promoter) and pMDC7 (under a β-estradiol-inducible G1090::XVE promoter) destination vectors by using LR Clonase enzyme (Life Technologies). These constructs were introduced into homozygous *sda1-1* mutant plants via *Agrobacterium*-mediated transformation using the floral dip method. Transgenic plants were screened for hygromycin resistance. The transformants in following generations were established as homozygous lines for further analysis. Supplementary Table S1 lists all of the primers used to confirm the positive transformants.

### RNA Isolation, Northern Hybridization, and RT-qPCR Analysis

Tissue samples were collected from soil-grown plants or seedlings grown on Peters/MS media plates (United Industries Corporation, United States) at the indicated time points, followed by flash-freezing in liquid nitrogen. Total RNA was isolated using TRIzol reagent according to manufacturer’s protocol (Invitrogen, Carlsbad, CA, United States). Northern blot analysis was performed as described previously (Dutta et al., 2017). RT-qPCR was used to check transcript levels of *SDA1* and *PR1* in selected plant lines. First-strand cDNA was synthesized using the SuperScript II RT Kit (Invitrogen). RT-qPCR reactions were performed in Bio-Rad CFX connect real time system using SYBR Green mix (Bio-Rad), as instructed by the manufacturer. All PCR reactions were performed in triplicates, normalized using internal control *UBC*, and averaged. For qPCR analysis. 2^–ΔΔCT^ method (Livak and Schmittgen, 2001) was used, and fold enrichments for WT genes were set to one. The data represent mean ± SEM of three independent biological replicates. All primers used in RT-qPCR analysis are listed in Supplementary Table S1.

### SA and SAG Measurements

For SA analyses, rosette leaves of 4-week-old plants of indicated genotypes were inoculated with 5 × 10^7^ cfu/mL *Pst* DC3000 and *Pst* DC3000 *(avrRpm1*). The tissue samples were collected at indicated time points and flash frozen. Free SA and SAG were extracted and quantified by HPLC as described previously (Jagadeeswaran et al., 2007).

### Abiotic Stress-Mediated Gene Expression Analysis

Abiotic stress treatment was essentially performed as described previously (Dutta et al., 2015). For salinity stress treatment, 7-day old seedlings were grown on MS media plates and transferred to Whatman paper soaked in 200 mM sodium chloride for 12 h. For heat treatment, 7-day old seedlings were transferred to 37°C constant temperature room for 24 h. For cold treatment, 7-day old seedlings were transferred to 4°C constant temperature incubator for 24 h. For drought treatment, 4-week-old plants were deprived of water for 10 days. Tissue samples were harvested following the above mentioned abiotic stress treatments and used for RNA blot analysis.

### Seed Germination and Root Growth Assays Under Oxidative Stress

Seed germination and root growth assays were essentially performed as described previously (Dutta et al., 2015). WT, *sda1-1*, *sda1-2*, pER8::*SDA1* transgenic lines, and amino acid mutant seeds were surface sterilized using 20% bleach for 5 min, followed by washing three times with sterile water in a sterile hood environment. Sterilized seeds were plated in MS medium in square petri plates. ROS-inducing agents (Paraquat, H_2_O_2_, NaCl, and 3-AT) were added to the MS medium. pER8::*SDA1* and amino acid mutant seeds were induced in the presence of 55 μm β-estradiol in MS media. These plates were assayed for percent-seed germination and compared with control MS medium (no treatment). Root growth was assayed with a digital camera (Nikon D70) and root length was calculated with ImageJ software. These experiments were repeated twice with similar results.

## Results

### Bacterial Pathogens Induce Expression of *SDA1*

**FIGURE 1:**
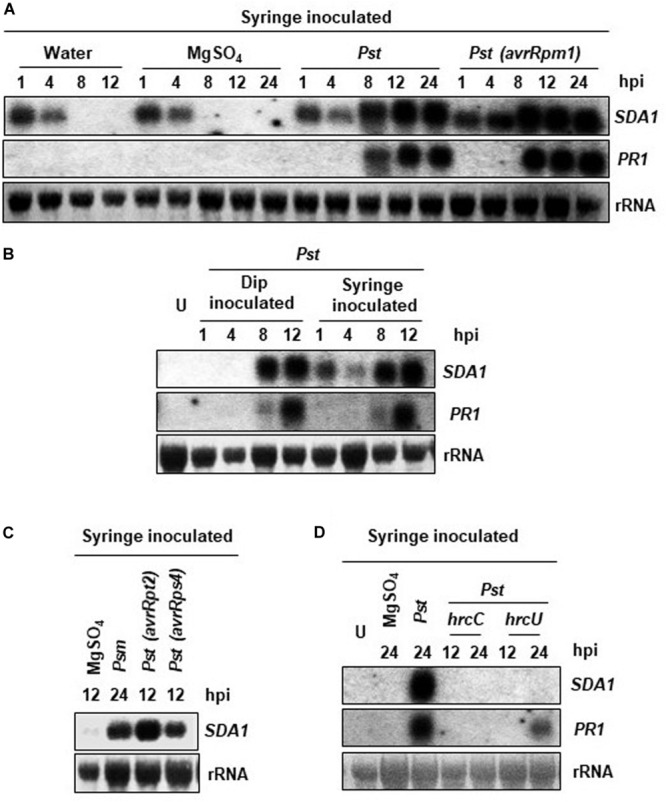
Expression analysis of the *SDA1* gene in response to bacterial pathogens. **(A–D)** Leaves of 4-week-old WT Col-0 plants were either syringe or dip inoculated with the indicated pathogens at a titer of 5 × 10^7^ cfu/mL in 10 mM MgSO_4_. Mock treatment was performed with 10 mM MgSO_4_. Tissue samples were harvested at the indicated hpi (hours post-inoculation). RNA was isolated and transcript levels were determined by RNA gel-blot analysis. Blots were stained with methylene blue to show the relative amounts of RNA in each lane (28S rRNA). Blots were probed with the *SDA1* gene probe, stripped and re-probed with the *PR1* gene probe. All experiments were repeated at least twice and similar results were obtained. *Pst, Pst* DC3000; *Psm, Psm* ES4326; *avrRpm1, Pst* DC3000 (*avrRpm1*); *avrRps4, Pst* DC3000 (*avrRps4*); *hrcC*, *hrp* mutant of *Pst* with a mutation in *hrcC* gene; *hrcU*, *hrp* mutant of *Pst* with mutation in *hrcU* gene; U, uninoculated.

SDA1 gene (At1g19020) was identified in a large microarray screen as a gene that is strongly induced in response to bacterial pathogens (Appel et al., 2014). In order to further understand the role of SDA1 in pathogen defense, we analyzed accumulation of *SDA1* transcript in wild-type Col-0 (WT) plants in response to bacterial pathogens. Rosette leaves of 4-week-old WT plants were inoculated with virulent bacterial pathogen *P. syringae* pv. *tomato* DC3000 (*Pst* DC3000) and avirulent bacterial pathogen *Pst* DC3000 expressing *avrRpm1* [*Pst* DC3000 (*avrRpm1*)], and accumulation of *SDA1* transcript was monitored by northern blot analysis. Control plants were infiltrated with 10mM MgSO_4_. In response to 10 mM MgSO_4_ (and distilled water), the *SDA1* gene was induced transiently within 1 h (Figure 1A). This transient expression of SDA1 could be the result of mechanical stress resulting from syringe infiltration. To test this possibility, we infected plants by dip inoculation method and analyzed the kinetics of SDA1 expression. There was no detectable induction of SDA1 at 1 and 4 h post-dipping, confirming that induction observed at 1 and 4 h was the result of mechanical stress induced by syringe infiltration (Figure 1B). Induction in response to *Pst* DC3000 started at 8 hours post-inoculation (hpi) and peaked by 12 hpi (Figure 1A). However, in response to *Pst* DC3000 (*avrRpm1*), SDA1 transcript started to accumulate as early as four hpi and peaked by eight hpi. This pattern of SDA1 expression in response to bacterial pathogens is similar to that of the PR1 gene, a widely used defense marker gene. However, SDA1 expression precedes PR1 gene expression. Expression of SDA1 was also induced in response to another virulent bacterial pathogen *P. syringae maculicola* ES4326 *(Psm*) and avirulent bacterial pathogens expressing *avrRpt2* or *avrRps4*, suggesting that SDA1 functions downstream of several R genes (Figure 1C).

Bacterial pathogens employ the type III secretion system (TTSS) to deliver the effector proteins into the host cells to cause disease. TTSS is encoded by the *hrp* genes; *hrcC* and *hrcU* mutants of *Pst* DC3000 are defective in delivering virulence factors into plant cells and therefore are non-pathogenic (Collmer et al., 2000). To determine if functional TTSS is required for expression of SDA1 during pathogen infection, we analyzed expression of SDA1 in response to *hrcC* and *hrcU* mutants. No SDA1 transcript was detected at 12 or 24 hpi by northern blot analysis suggesting that the transfer of effector proteins is required for expression of SDA1 (Figure 1D).

### Pathogen-Induced *PR* Gene Expression Is Compromised in *sda1* Mutants

**FIGURE 2:**
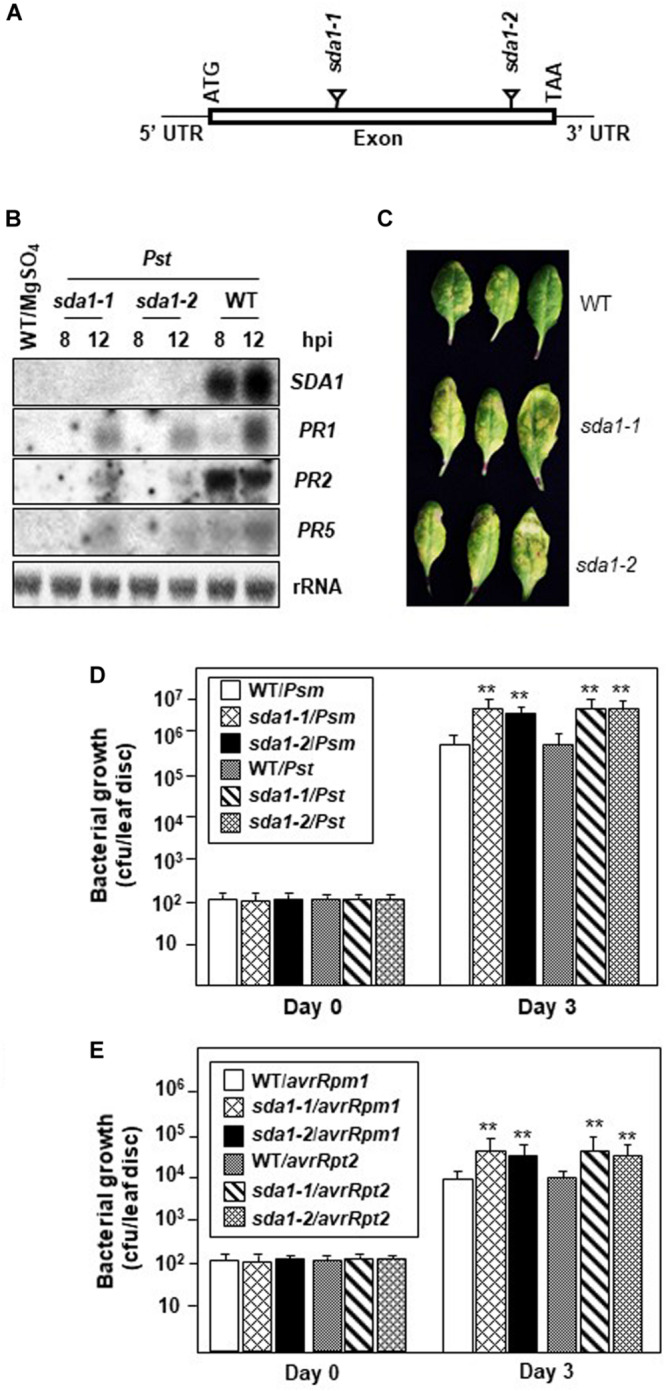
*PR* gene expression and resistance against bacterial pathogen is compromised in *sda1* mutants. **(A)** Diagram depicting site of insertion of T-DNA in *sda1-1* and *sda1-2* mutants. The Rectangle indicates exon and black lines indicate 5’ and 3’ UTRs. **(B)** Total RNA was isolated from the leaves of 4-week-old plants of indicated genotypes inoculated with virulent bacterial pathogen *Pst* DC3000 (*Pst*) at a titer of 5 × 10^7^ cfu/mL in 10 mM MgSO_4_. Tissue samples were harvested at indicated hpi. Transcript levels were determined by RNA gel-blot analysis. Blots were stained with methylene blue to show the relative amounts of RNA in each lane (28S rRNA). Blots were probed with *SDA1* gene probe, stripped and re-probed with *PR* gene probes as indicated. **(C)** Rosette leaves of 4-week-old plants of indicated genotypes were inoculated with *Pst* DC3000 at a titer of 5 × 10^5^ cfu/mL and leaves were photographed at 3 dpi (days post-inoculation). **(D,E)** Rosette leaves of 4-week-old plants of the indicated genotypes were inoculated with indicated pathogen at a titer of 5 × 10^5^ cfu/mL. Eight plants for each genotype were analyzed individually. Data are reported as mean bacterial count (cfu per leaf disc) ± SD. Asterisks indicate statistically significant differences Statistical analysis was performed by one-way ANOVA followed by Dunnett’s test with reference to WT control. Asterisks indicate statistically significant differences (**p < 0.01). This experiment was repeated two more times with similar results. *Psm, Psm* ES4326; *Pst, Pst* DC3000; *avrRpm1, Pst* DC3000 expressing *avrRpm1; avrRpt2, Pst* DC3000 expressing *avrRpt2*.

Defense against pathogens is usually associated with activation of PR genes. To determine if SDA1 is involved in activation of defense genes in response to pathogen infections, we determined expression of PR1 in *sda1* mutants in response to bacterial pathogens. We identified two T-DNA insertion lines, *sda1-1* (SAIL_390_F08, Syngenta Arabidopsis Insertion Library collection) (Sessions et al., 2002) and *sda1-2* (GABI_283D01, GABI-Kat T-DNA insertional collection) (Rosso et al., 2003; Figure 2A). Sites of T-DNA insertion in these lines were confirmed by determining the sequence of the genomic DNA flanking the T-DNA. The T-DNA was inserted between nucleotides 85 and 86 of the open reading frame in *sda1-1* and between nucleotides 149 and 150 of the open reading frame in *sda1-2*. To determine if these T-DNA insertion lines represent null alleles of *SDA1*, expression of *SDA1* in response to *Pst* DC3000 was analyzed in these lines by northern blot analysis. No transcript was detected, confirming that these lines represent null alleles of *SDA1* (Figures 2A,B). No visible phenotypic differences were observed in the *sda1* mutants compared to the WT plants. Expression of *PR* genes in *sda1* mutant plants was analyzed 8 and 12 hpi by northern blot analysis. As expected, the *PR1* gene was strongly induced in the WT plants in response to *Pst* DC3000. However, expression in both *sda1* mutants was significantly compromised and delayed. Similarly, expression of two other defense genes, *PR2* and *PR5* was also compromised in *sda1* mutants (Figure 2B). Taken together, these results suggest that full pathogen induction of *PR1*, *PR2*, *PR5*, and likely other defense-related genes requires SDA1.

### Defense Against Bacterial Pathogens Is Compromised in *sda1* Mutants

To determine the role of SDA1 in defense against bacterial pathogens, we assessed the growth of virulent and avirulent bacterial pathogens in the *sda1* knockout lines. Virulent *Pst* DC3000 was inoculated into the rosette leaves of *sda1* and WT plants, and development of disease symptoms and growth of the pathogen was monitored for 3 days post-inoculation (dpi). *sda1* mutants developed more disease symptoms and supported ∼5–7-fold more pathogen growth compared to the wild-type. Similar results were obtained with another virulent pathogen *Pseudomonas syringae* pv. *maculicola* ES4326 (*Psm)* (Figures 2C,D). These results suggest that SDA1 is required for basal resistance against a variety of virulent bacterial pathogens.

To test whether *sda1* mutants are compromised in *R* gene-mediated resistance (ETI), rosette leaves of 4-week-old *sda1* mutants were inoculated with *Pst* DC3000 expressing *avrRpm1* [*Pst* DC3000 (*avrRpm1*)] or *avrRpt2* [*Pst* DC3000 (*avrRpt2*)], and pathogen growth was determined 3 dpi. Both avirulent pathogens grew ∼3–4-fold more in the *sda1* mutants compared to the WT (Figure 2E). These results suggest that SDA1 also contributes to *R* gene-mediated resistance.

### *SDA1* Overexpression Induces *PR* Genes

To further determine the role of SDA1 in pathogen defense, we constructed transgenic plants expressing *SDA1* under the control of a β-estradiol-inducible XVE system (pER8::*SDA1*) in the pMDC7 vector (Zuo et al., 2000; Curtis and Grossniklaus, 2003). Upon treatment with β-estradiol, these lines accumulated *SDA1* transcript in an β-estradiol concentration-dependent manner (Supplementary Figure S1A). The resulting elevated levels of *SDA1* transcript correlated with increasing *PR* gene expression (Supplementary Figure S1A). Together, these results suggest that SDA1 positively modulates expression of *PR* genes and likely other defense genes. To assess whether overexpression of *SDA1* would confer enhanced resistance against virulent bacterial pathogens, growth of *Pst* DC3000 in these lines was tested. Pathogen growth was reduced ~5–7-fold in the *SDA1* inducible overexpression lines compared to that in the WT plants (Supplementary Figure S1B).

### Expression of *SDA1* Is Modulated via the Salicylic Acid Signaling Pathway

**FIGURE 3:**
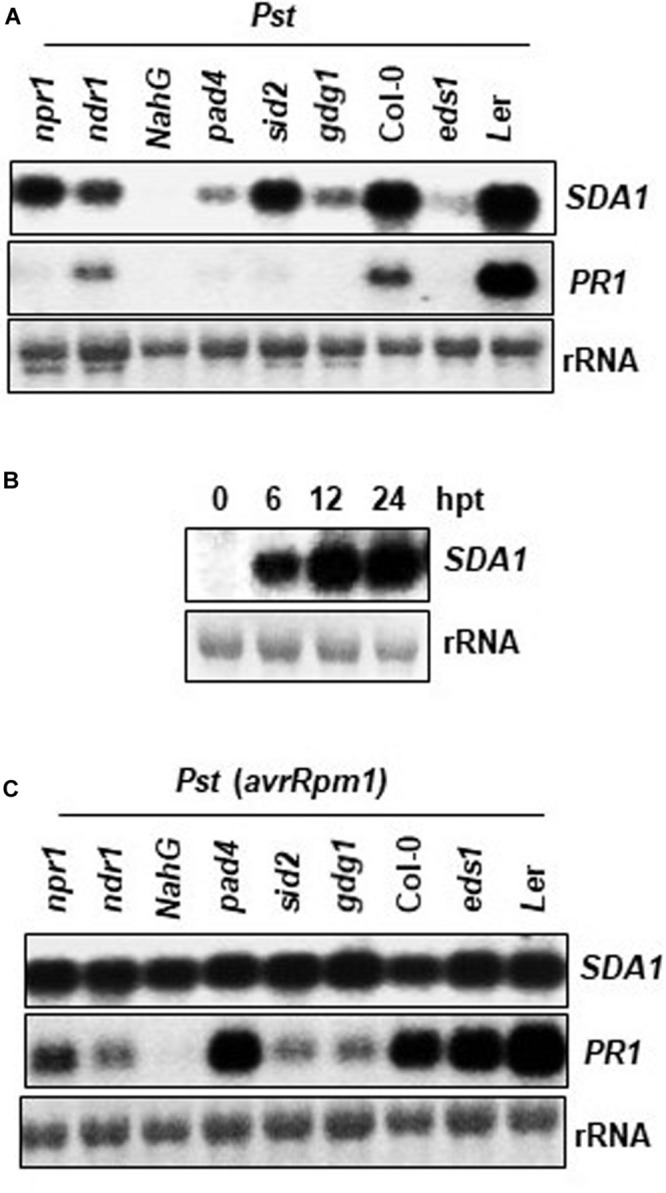
Expression analysis of *SDA1* in defense mutants and in response to SA. **(A,C)** Mutant and wild type plants (4-week-old) were treated with *Pst* DC3000 **(A)** and *Pst* DC3000 *(avrRpm1)* **(C)** at a concentration of 5 × 10^7^ cfu/mL and tissue samples were harvested at 24 hpi (hours post-inoculation). Northern gel blot analysis was employed to determine transcript levels. Blots were stained with methylene blue to show the relative amounts of RNA in each lane (rRNA). Blots were probed with *SDA1* gene probe, stripped and re-probed with *PR1* gene probe. All mutants are in the Col-0 background, except *eds1*, which is in the *Ler* background. **(B)** Four-week-old wild-type (Col-0) plants were sprayed with 1 mM SA. Rosette leaves were collected at the indicated hours post-treatment (hpt) for RNA isolation. Transcript levels were determined by RNA gel-blot analysis. Blots were stained with methylene blue to show the relative amounts of RNA in each lane (28S rRNA). Blots were probed with the *SDA1* gene probe. All experiments were repeated at least twice and similar results were obtained. *Pst*, *Pst* DC3000; *Pst* (*avrRpm1*), *Pst* DC3000 (*avrRpm1*).

To determine the epistatic relationship of SDA1 with other components of the defense-signaling network, we analyzed expression of *SDA1* in response to *Pst* DC3000 in several Arabidopsis mutants compromised in defense against pathogens. Rosette leaves of 4-week-old WT and mutant plants were inoculated with *Pst* DC3000 and expression of *SDA1* was determined 24 hpi. Expression of *SDA1* was compromised in *pad4* (Glazebrook et al., 1996), *eds1* (Parker et al., 1996), *pbs3/gdg1/win3* (Jagadeeswaran et al., 2007; Lee et al., 2007; Nobuta et al., 2007), and *ndr1* (Century et al., 1995) mutants (Figure 3A). In contrast, mutation in *sid2* (Wildermuth et al., 2001) and *npr1* (Cao et al., 1994) had negligible effect on *SDA1* expression. Taken together, these results suggest that SDA1 is downstream of PAD4, EDS1, GDG1 and NDR1 but is either upstream of SID2 and NPR1 or is in another signaling pathway. However, epistatic analysis across mutants of each indicated gene (or groups of genes) will be required to establish these relationships. Furthermore, expression of *SDA1* was blocked in *NahG* plants, suggesting that SA is required for expression of *SDA1*. To test if SA is sufficient to induce the expression of *SDA1*, SA was applied externally to the WT plants. Expression of *SDA1* was induced within 6 h, and peaked by 12 h after SA application, suggesting that SA alone is sufficient to induce expression of *SDA1* (Figure 3B). Expression of *SDA1* was also assessed in these mutants in response to *Pst* DC3000 (*avrRpm1*) but no change in *SDA1* expression was detected (Figure 3C). These results suggest that *R* gene-mediated induction of *SDA1* is independent of the SA-mediated defense pathway or that in response to avirulent pathogens signals other than SA may be involved in *SDA1* activation. Alternatively, the threshold level of SA-mediated signaling required to activate *SDA1* expression may be sufficiently low such that the robust *R* gene-mediated signal, despite being compromised by the various mutants, is still above this threshold.

### SA Rescues Defense Responses in *sda1* Mutants

Resistance to biotrophic bacterial pathogens is typically dependent on SA. Mutants compromised in SA biosynthesis (e.g., *sid2*), metabolism (e.g., *pbs3/gdg1/win3*), or signaling (e.g., *npr1*) are compromised in resistance against a variety of bacterial, fungal and viral pathogens. To determine if *sda1* mutants are compromised in SA signaling, *PR1* expression was assessed in *sda1* mutants in response to external application of SA. *PR1* expression was induced to similar levels in the *sda1* mutant and the WT plants (Supplementary Figure S2A). Additionally, an application of SA also rescued resistance against *Pst* DC3000 (Supplementary Figure S2B) in *sda1* mutants. Together these results demonstrate that SA-mediated signaling is not compromised in the *sda1* mutants.

### SA Accumulation in Response to Pathogen Infection Is Impaired in *sda1* Mutants

**FIGURE 4:**
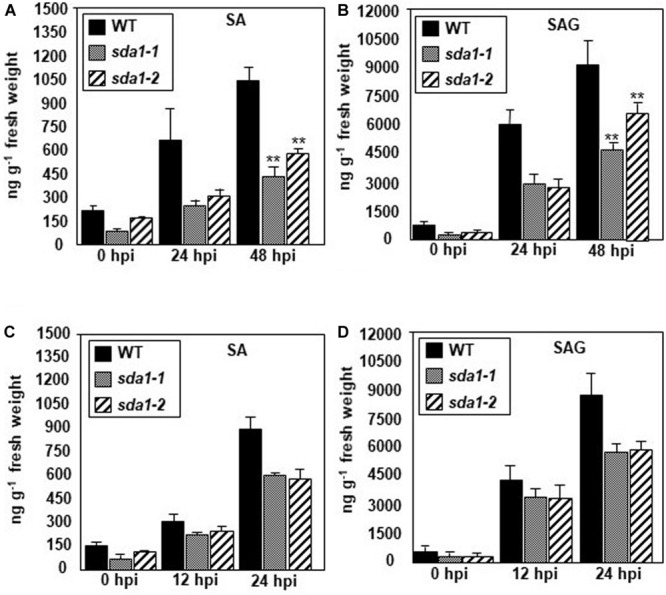
*sda1* mutant is compromised in accumulating pathogen-inducible SA and SAG. Rosette leaves of 4-week-old plants of wild-type (WT) and *sda1* mutants were inoculated with *Pst* DC3000 **(A,B)** and *Pst* DC3000 *(avrRpm1)* **(C,D)** at a titer of 5 × 10^7^ cfu/mL in 10 mM MgSO_4_. Tissue samples were collected at the indicated hpi and free SA (SA) and glucose-conjugated SA (SAG) were extracted. The values are means ± SD of three replicates, each consisting of leaves from 10 plants per genotype. Statistical analysis was performed by one-way ANOVA followed by Dunnett’s test with reference to WT control. Asterisks indicate statistically significant differences (**p < 0.05).

The above results suggest that SA perception and signaling is not impaired in *sda1* mutants. To determine if SDA1 has any role to play in the accumulation of SA in response to pathogens, we determined levels of free SA and SA glucoside (SAG) in *sda1* mutants in response to *Pst* DC3000 (Figures 4A,B) and *Pst* DC3000 (*avrRpm1*) (Figures 4C,D). Accumulation of free SA and SAG in response to *Pst* DC3000 was reduced ∼2–3-fold in *sda1* mutants compared to the WT plants (Figure 4). The reduced levels of SA and SAG accumulation in response to infection by *Pst* DC3000 might explain the enhanced susceptibility of *sda1* mutants to virulent *P. syringae.*

### *SDA1* Is Induced in Response to Reactive Oxygen Species

Accumulation of reactive oxygen species (ROS) is one of the earliest plant defense responses against pathogen infection (Torres et al., 2006). ROS function as antimicrobial compounds and also act as signaling molecules for defense activation. To determine if ROS might play a role in activation of *SDA1*, we determined the expression of *SDA1* in response to H_2_O_2_, 3-AT (3-amino-1,2,4-triazole), and paraquat (methyl viologen, N,N’-Dimethyl-4,4’-bipyridinium dichloride) over a 48 h period by northern blot analysis. 3-AT is an irreversible inhibitor of catalase, which scavenges H_2_O_2_ from the photorespiration system in leaves, and thus an application of 3-AT results in accumulation of intracellular H_2_O_2_ (Wang et al., 1999). Paraquat is widely used as source of intracellular superoxide radicals (Babbs et al., 1989). Treatment with H_2_O_2_ resulted in a rapid but very transient expression of *SDA1.* This is consistent with the previous report that *SDA1* is induced in response to H_2_O_2_ (Davletova et al., 2005). In contrast, 3-AT induced a slower but prolonged induction of *SDA1* expression; while *SDA1* induction by paraquat was more modest and not as prolonged as with 3-AT (Supplementary Figure S3A). Together these results suggest that activation of SDA1 in response to pathogen might be modulated via intracellular ROS.

Several lesion mimic mutants constitutively express defense-related genes, accumulate high levels of ROS, and have enhanced resistance against several virulent pathogens. We determined expression of *SDA1* in some of these lesion mimic mutants by northern blot analysis. All tested lesion mimic mutants *hrl1*, *acd2*, *dll1*, and *lsd1* (Dietrich et al., 1994; Greenberg et al., 1994; Devadas et al., 2002; Pilloff et al., 2002; Dutta et al., 2015) accumulate high levels of *SDA1* transcript indicating that high levels of ROS in these mutants might be responsible for activation of *SDA1* (Supplementary Figure S3B).

### *SDA1* Is Induced in Response to Abiotic Stresses

Whole genome expression analysis has indicated induction of *SDA1* in response to multiple abiotic stresses (Davletova et al., 2005; Dinneny et al., 2008; Luhua et al., 2008). To determine if SDA1 might play any role in modulating abiotic stress in a temporal context, we determined expression of *SDA1* in response to salinity, drought, cold stress and heat stress. Salt, cold and heat stress treatments were performed on 7-day-old seedlings, and drought treatment was performed on 4-week-old-plants as described in the Experimental Procedures. All tested stress treatments induced expression of SDA1 (Supplementary Figures S3C,D). Taken together, these results suggest that SDA1 might be a stress response protein involved in modulating both biotic and abiotic stresses.

### Temporal and Spatial Expression of *SDA1*

**FIGURE 5:**
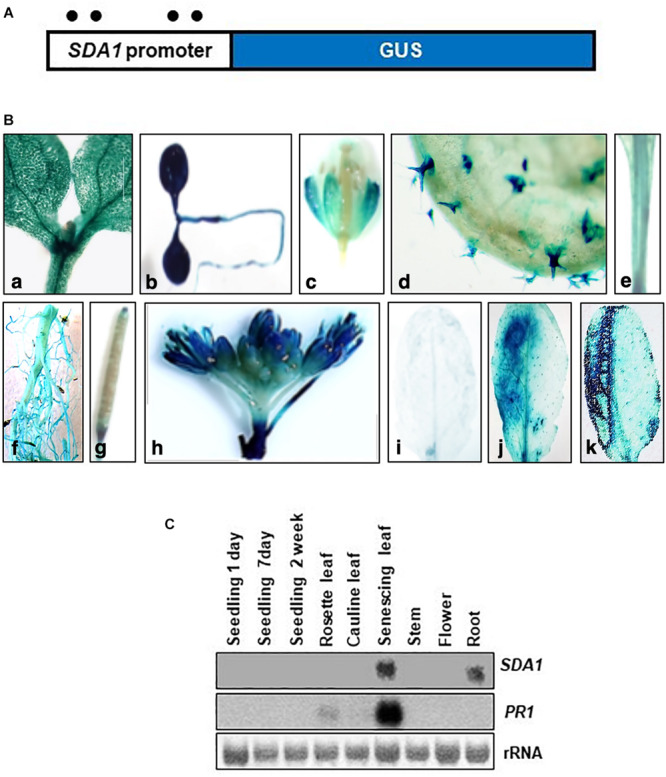
Histochemical analysis of GUS expression in transgenic plants expressing the *SDA1 promoter*::GUS construct. **(A)** Schematic representation of the *SDA1 promoter*::GUS fusion construct. A 2 kb promoter region of the *SDA1* gene was used to drive expression of the GUS gene. Filled circles show position of W-boxes. **(B)** GUS expression in transgenic plants expressing the *SDA1 promoter*::GUS: (a,b) 10-day old seedling, (c) flower, (d) trichome, (e) stem, (f) root, (g) silique, (h) inflorescence, (i) untreated rosette leaf, (j) rosette leaf inoculated (left half) with *Pst* DC3000 at titer of 5 × 10^7^ cfu/mL, (k) rosette leaves wounded (left half with serrated forceps). This analysis was carried out in five to seven independent transgenic lines and similar results were observed. **(C)** Northern gel blot analysis was employed to determine transcript levels of *SDA1* in indicated tissue samples. Blot was stained with methylene blue to show the relative amounts of RNA in each lane (rRNA) and was hybridized with indicated gene probes.

To analyze temporal and spatial expression of *SDA1*, we constructed transgenic plants containing *SDA1* promoter::*GUS* fusion construct. A 2000 bp region containing the 5‘ UTR region of *SDA1* was fused with the β-glucuronidase (*GUS*) gene and was used to transform wild-type plants (Figure 5A). GUS analysis was carried out during various stages of plant development. *SDA1* promoter activity was detected in 10-day old seedlings, flowers, trichomes, stem, root, and at the tip of the siliques (Figures 5Ba–h). Faint GUS activity was also detected in rosette and cauline leaves of 4-week-old plants. These results suggest that SDA1 is developmentally regulated. To determine the spatial response of the *SDA1* promoter, rosette leaves of these plants were inoculated with *Pst* DC3000. GUS staining was detected 12 hpi and was limited to the region of infiltration (Figures 5Bi,j). In addition, consistent with results that *SDA1* is induced by mechanical injury (see Figure 1B); strong GUS activity was detected at the site on wounding (Figure 5Bk). This is consistent with the gene expression analysis results in Figures 1A,B.

Expression of *SDA1* in these tissues and in senescing leaves was also analyzed by northern blot analysis. *SDA1* transcript was detected only in roots and in senescing leaves suggesting that transcript levels in seedlings, leaves, stems, and flowers are not high enough to be detected by northern blot analysis. Furthermore, since senescence is a type of programmed cell death, expression of *SDA1* in senescing leaves suggests that SDA1 might have a role in programmed cell death as well. Consistent with previous reports, *PR1* expression was also detected in senescing leaves (Figure 5C). Analysis of the 2 kb upstream region of *SDA1* for stress and pathogen responsive elements identified four W-boxes (TTGAC) (Figure 5A). W-boxes are the binding sites for WRKY transcription factors known to modulate defense-related genes (Pandey and Somssich, 2009).

### *SDA1* Encodes a Small Plant-Specific Protein

**FIGURE 6:**
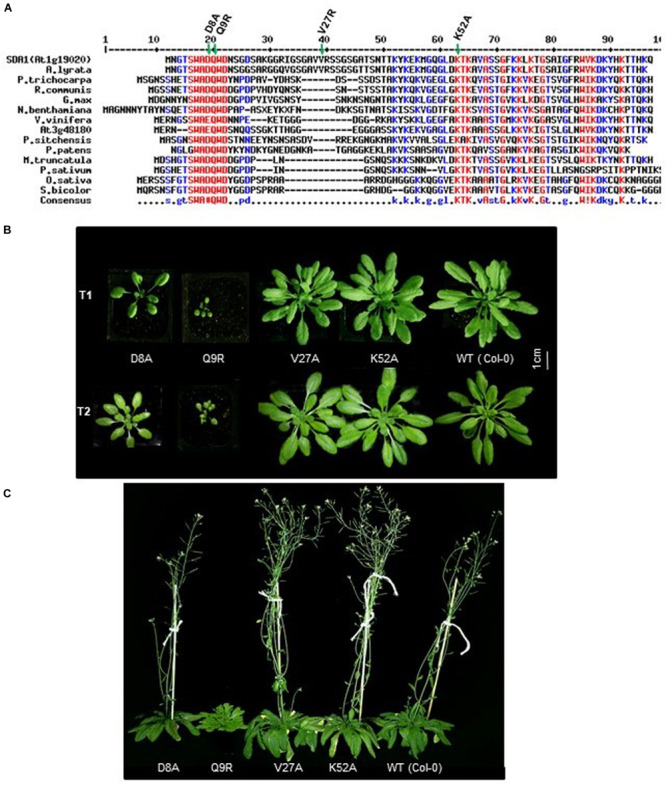
Conserved D8 and Q9 amino acid of SDA1 are functionally important for proper growth. **(A)** Alignment of Small Defense-Associated (SDA) protein family members in plants. An Alignment was done using MultAlin. Conserved (S/G)WA(D/E)QWD domain is underlined. All engineered amino acid mutants are indicated by asterisks. *Arabidopsis lyrate* (related to Arabidopsis), *Populus trichocarpa* (black cottonwood), *Ricinus communis* (castor bean), *Glycine max* (soybean), *Nicotiana benthamiana* (related to tobacco), *Vitis vinifera* (common grape vine), *Picea sitchensis* (sitka spruce), *Physcomitrella patens* (spreading earthmoss), *Medicago truncatula* (barrelclover), *Pisum sativum* (pea), *Oryza sativa* (asian rice), *Sorghum bicolor* (sorghum). **(B)** Growth of T_1_ and T_2_ generation of SDA1 D8A, Q9R, V27A, K52A mutant and WT (wild type) Col-0 plants. Plants were photographed at 25 days after sowing in day neutral light (12 h light:12 h dark). **(C)** Flowering of SDA1 D8A, Q9R, V27A, K52A mutant and WT Col-0 plants. Plants are photographed at 70 days after sowing (DAS) in day neutral light (12 h light: 12 h dark).

*SDA1* (At1g19020) is predicted to encode a small protein of 86 amino acids (calculated mass 9.15 kDa, pI 10.17). The Arabidopsis genome contains a homolog of *SDA1*, At3g48180 (*SDA2*), which is predicted to encode a 77 amino acid-long protein that shares 52% identity and 63% similarity with *SDA1*. Homologs of *SDA1* are present in several plant species but no other organism (Figure 6A). All these proteins have a highly conserved novel seven amino acid (S/G)WA(D/E)QWD domain at the N-terminus (Figure 6A). To the best of our knowledge, this domain has not been previously reported. Given that the founding member of this family, SDA1, is involved in pathogen defense, we call this family “small defense-associated (SDA)” protein family and the seven-amino acid domain, the “SWAD” domain.

In order to determine if the SWAD domain is critical for SDA1 function, we generated four point-mutant constructs by site-directed mutagenesis. Two of these mutations were introduced in the SWAD domain to change aspartic acid at position 8 to alanine (SDA1^D8A^), and glutamine at position 9 to arginine (SDA1^Q9R^). The third mutation was introduced in one of the conserved lysine residues at position 52 to change it to alanine (SDA1^K52A^), and the fourth one was introduced in a non-conserved valine at position 27 to change it to alanine (SDA1^V27A^) (Figure 6A). A constitutive 35S promoter was used to drive the expression of these engineered constructs and they were introduced into WT plants. For each construct, we analyzed 18 independent transgenic lines. Nine 35S::*SDA1*^D8A^ transgenic lines were mildly compromised in their overall growth compared to wild-type plants. Ten 35S::*SDA1*^Q9R^ transgenic lines were severely stunted and were significantly delayed in flowering (Figures 6B,C). Subsequently, these stunted lines had a poor seed set and some of them failed to set any seeds at all. All 35S::*SDA1*^K52A^ and 35S::*SDA1*^V27A^ transgenic lines grew normally and showed no visible phenotypic differences compared to WT. These developmental phenotypes of 35S::*SDA1*^D8A^ and 35S::*SDA1*^Q9R^ transgenic lines are likely due to dominant negative effects of these mutations.

### 35S::*SDA1*^D8A^ and 35S::*SDA1*^Q9R^ Plants Are Compromised in Defense Against *Pst* DC3000

To determine if the SWAD domain has any role to play in modulating SDA1-mediated defense in Arabidopsis, we analyzed transcript levels of *SDA1* and *PR1* by reverse transcriptase-quantitative PCR (RT-qPCR) in two constitutively overexpressing independent transgenic lines of each of the amino acid mutants (Supplementary Figure S4A). While *SDA1* levels were relatively similar in all the transgenic lines, *PR1* levels were significantly reduced only in 35S::*SDA1*^D8A^ and 35S::*SDA1*^Q9R^ transgenic lines. These results suggest that aspartic acid at position 8 and glutamine at position 9, and most likely the entire SWAD domain is required for SDA1-mediated expression of *PR1* and most likely other defense genes.

**FIGURE 7:**
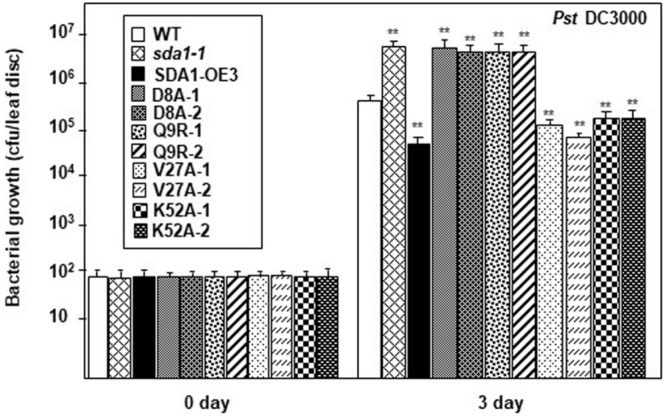
SDA1 D8A and Q9R amino-acid mutants are susceptible against *Pst* DC3000. Rosette leaves of 4-week-old plants were treated with 55 μm of β-estradiol and 24 h later the leaves were inoculated with *Pst* DC3000 at a titer of 5 × 10^5^ cfu/mL and pathogen growth was determined at indicated days after inoculation. Eight plants for each genotype were analyzed individually. Data are reported as mean bacterial count (cfu per leaf disc) ± SD. Statistical analysis was performed by one-way ANOVA followed by Dunnett’s test with reference to WT control. Asterisks indicate statistically significant differences (**p < 0.01). Statistical analysis is with reference to WT. This experiment was repeated two more times with similar results. SDA1-OE3, *SDA1* expressed from β-estradiol-inducible XVE system (pER8::*SDA1*, line # 3); D8A, Q9R, V27A, K52A are various point mutants of *SDA1* expressed from pER8 promoter.

As described above (Figure 6), 35S::*SDA1*^D8A^ and 35S::*SDA1*^Q9R^ transgenic lines have severe developmental defects, and therefore are not suitable for bacterial pathogen growth assays (especially 35S::*SDA1*^Q9R^). Therefore, in order to determine if the SWAD domain has any role in defense against pathogens, we constructed transgenic lines expressing *SDA1* point mutants from an β-estradiol-inducible promoter (pER8::*SDA1*^D8A/Q9R/V27A/K52A^) in the *sda1-1* mutant background. In the absence of external β-estradiol, no transgenic line showed any visible phenotype. In response to β-estradiol application, all transgenic lines induced expression of the transgene (Supplementary Figure S4B). However, expression of *PR1* was significantly induced only in transgenic lines expressing pER8::*SDA1*^K52A^ and pER8::*SDA1*^V27A^ but not in the transgenic lines expressing pER8::*SDA1*^D8A^ and pER8::*SDA1*^Q9R^ (Supplementary Figure S4B). These results are consistent with the expression of *PR1* in transgenic lines expressing *SDA1* point mutants from the constitutive 35S promoter (Supplementary Figure S4A). To test for response of these transgenic lines to virulent bacterial pathogens, 4-week-old rosette leaves of WT, *sda1-1*, mutant lines expressing β-estradiol-inducible transgenes, and pER8::*SDA1* were induced by β-estradiol. After 24 h, all plants were inoculated with *Pst* DC3000 and pathogen growth was determined 3 dpi. Pathogen growth in pER8::*SDA1*^K52A^ and pER8::*SDA1*^V27A^ transgenic lines was similar to that in the pER8::*SDA1* line, however, growth in the pER8::*SDA1*^D8A^ and pER8::*SDA1*^Q9R^ transgenic lines was similar to the *sda1* mutant (Figure 7). These results suggest that the SWAD domain is critical for SDA1-mediated resistance against pathogens. Together, these results suggest that conserved amino acids aspartic acid at position 8, glutamine at position 9, and possibly the entire SWAD domain are functionally important for SDA1 function in defense against bacterial pathogen *Pst* DC3000.

### *SDA1* Modulates Tolerance Against Oxidative Stress

**FIGURE 8:**
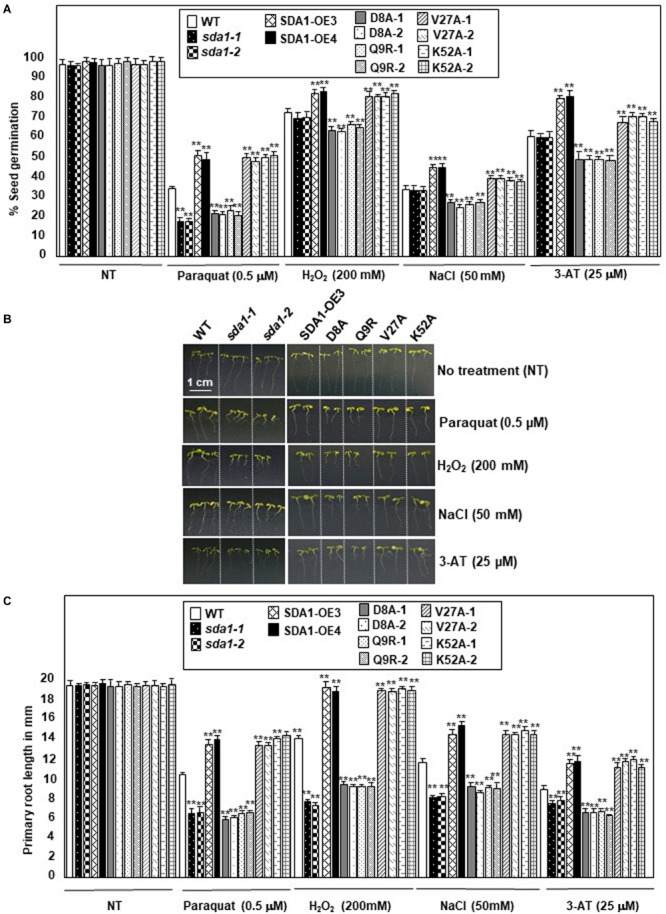
*SDA1* is required for tolerance to oxidative stress. **(A)** Percent seed germination of WT, *sda1* mutants, pER8::*SDA1* plants, and various amino acids mutants of *SDA1* expressed from pER8 promoter in the presence of oxidative stress inducing chemicals. **(B)** Representative 5-days-old seedlings of genotypes in **(A)** grown in the presence of indicated oxidative stress inducing chemicals. Scale bar is 1 cm for all samples. **(C)** Mean root length of seedlings in **(B)**. (*n* = 25–35; Student’s *t*-test; **p < 0.01). Statistical analysis was performed by one-way ANOVA followed by Dunnett’s test with reference to respective WT control of each treatment. Asterisks indicate statistically significant differences (**p < 0.01). All experiments were repeated twice and similar results were obtained. SDA1-OE3 and SDA1-OE4, *SDA1* expressed from β-estradiol-inducible XVE system (pER8::*SDA1*, line # 3 and # 4); D8A, Q9R, V27A, K52A are various point mutants of *SDA1* expressed from pER8 promoter.

The enhanced *SDA1* expression in response to oxidative stress suggested that SDA1 may be involved in modulating tolerance to oxidative stress. Oxidative stress has been shown to affect both seed germination (Bailly et al., 2008) and seedling root growth (Tsukagoshi, 2016) in Arabidopsis. We analyzed seed germination of *sda1* mutants in the presence of ROS-producing agents including paraquat (Apel and Hirt, 2004), H_2_O_2_ (Apel and Hirt, 2004), NaCl (Borsani et al., 2001), and 3-AT (Ozgur et al., 2015). Under normal growth conditions, seed germination and seedling root growth of *sda1* mutants were not significantly different from the WT (Figure 8 and Supplementary Figure S5). However, when subjected to exogenous oxidative stress, *sda1* seeds germinate poorly in the presence of paraquat but not H_2_O_2_, NaCl and 3-AT (Figure 8A and Supplementary Figure S5A). Furthermore, germination rate of two independent lines of pER8::*SDA1* was higher than the WT and *sda1* mutants in the presence of all oxidative stress generating agents (Figure 8A). Seed germination of pER8::*SDA1*^K52A^ and pER8::*SDA1*^V27A^ transgenic lines was similar to the pER8::*SDA1* lines, however, germination of pER8::*SDA1*^D8A^ and pER8::*SDA1*^Q9R^ transgenic lines was comparable to the *sda1* mutant.

We also tested the effect of these oxidative stress conditions on root growth for *sda1*, pER8::*SDA1*, and pER8::*SDA1* point-mutant transgenic lines. Roots of *sda1* mutants grew significantly shorter compared to the WT in the presence of paraquat, H_2_O_2_, and NaCl (Figures 8B,C and Supplementary Figure S5B). A more modest but statistically significant difference in root length was observed in the *sda1* mutant in the presence of 3-AT compared to the WT. Furthermore, pER8::*SDA1* lines grew longer roots in presence of oxidative stress than WT (Figures 8B,C). Similar to the seed-germination phenotype, root-length of pER8::*SDA1*^D8A^ and pER8::*SDA1*^Q9R^ transgenic lines was comparable to the *sda1* mutant. However, root-length of pER8::*SDA1*^K52A^ and pER8::*SDA1*^V27A^ transgenic lines was similar to pER8::*SDA1* lines. Consistent with our results that overexpression of *SDA1* confers resistance to oxidative stress, a previous paper reported that overexpression of *SDA1* leads to sensitivity to salt stress (Luhua et al., 2008). The reason for this discrepancy to salt stress is not known at this time. Nevertheless, taken together, these results suggest a role for SDA1 and its SWAD domain in tolerance to oxidative stress.

## Discussion and Conclusion

Here, we report the characterization of the SDA1 gene of Arabidopsis (*Arabidopsis thaliana*). Several lines of evidence suggest that SDA1 is required for resistance against the bacterial pathogen *P. syringae*. First, SDA1 is induced rapidly and to high levels in response to several virulent and avirulent strains of *P. syringae*. Like a typical defense gene, SDA1 induction in response to avirulent strain is faster and to higher levels compared to the virulent strains. Second, pathogen-mediated induction of SDA1 requires functional TTSS, suggesting that virulence factors must be delivered into the plant cells for induction of SDA1. Third, pathogen-mediated induction of SDA1 is compromised in several defense mutants. Finally, induction of defense genes and resistance against virulent and avirulent strains of *Pst* DC3000 is compromised in *sda1* knockout mutants.

*sda1* mutation compromises resistance against virulent pathogens more significantly than resistance against avirulent pathogens. This may be the result of a significantly higher loss in SA/SAG accumulation in *sda1* mutant in response to virulent pathogens as compared to avirulent pathogens. Furthermore, while expression of *SDA1* is significantly compromised in defense signaling mutants in response to virulent pathogen, it is not affected in response to avirulent pathogen. This could be because the threshold level of SA-mediated signaling required to activate *SDA1* expression may be sufficiently lower than that required for robust *R* gene-mediated signaling.

SDA1 is predicted to code for a small 86-amino acid protein. It does not have homology to any protein of known function in the public databases. The Arabidopsis Information Resource (TAIR, Araport 11) indicates predicted function as a CDP-diacylglycerol-glycerol-3-phosphate 3-phosphatidyltransferase, an enzyme that participates in glycerophospholipid metabolism. Glycerophospholipids have been described as key regulators of stress response and salicylic acid signaling (Janda et al., 2013; Janda and Ruelland, 2015; Gujas and Rodriguez-Villalon, 2016; Verma and Agrawal, 2017). Homologs of SDA1 are present in several plant species including bean, grape, poplar, tobacco, medicago (alfalfa), soybean and rice. However, no homologs in other plant species or any other organism have been reported suggesting that SDA1 is a plant-specific protein. All homologs of SDA1 have a novel small seven amino acid (S/G)WA(D/E)QWD conserved domain at the N-terminus of the protein; this may suggest that this domain is critical for protein function. We identified critical roles for the conserved domain in SDA1 function specifically for bacterial defense and ROS tolerance, which further highlights the importance of this novel domain.

In addition to pathogens, expression of SDA1 is also induced in response to a variety of biotic and abiotic stresses, including aphid (Myzus persicae), insect larvae (Spodoptera), and oxidative stress induced by glucose-glucose oxidase (Appel et al., 2014). *SDA1* expression is also induced in response to abiotic stresses such as drought, salt, heat, wounding, and ROS-generating chemicals. All these abiotic stresses are known to generate ROS. *sda1* mutants are hypersensitive while overexpressing pER8::*SDA1* lines are more tolerant to oxidative stress-inducing agents such as paraquat, H_2_O_2_, NaCl, 3-AT, which suggests a role in oxidative stress tolerance. Overall, these results suggest that SDA1 functions as a broad-range, stress–response plant protein.

*PR* gene expression is delayed and compromised in *sda1* mutants in response to virulent bacterial pathogen *Pst* DC3000. External application of SA rescues both *PR1* expression and resistance to pathogens in *sda1* mutants. These results suggest that the *sda1* mutant is not compromised in sensing or signaling mediated by SA and that SDA1 probably functions upstream of SA. Additionally, accumulation of SA and SAG in response to *Pst* DC3000 is compromised in *sda1* mutants, further suggesting that SDA1 acts upstream of SA. However, external application of SA induces expression of SDA1, suggesting a SA-mediated positive amplification loop that might modulate expression of SDA1 in response to pathogen infections. Similar SA-mediated positive amplification loops have been proposed previously for other defense genes, such as *PAD4* and *GDG1* (Jirage et al., 1999; Jagadeeswaran et al., 2007).

**FIGURE 9:**
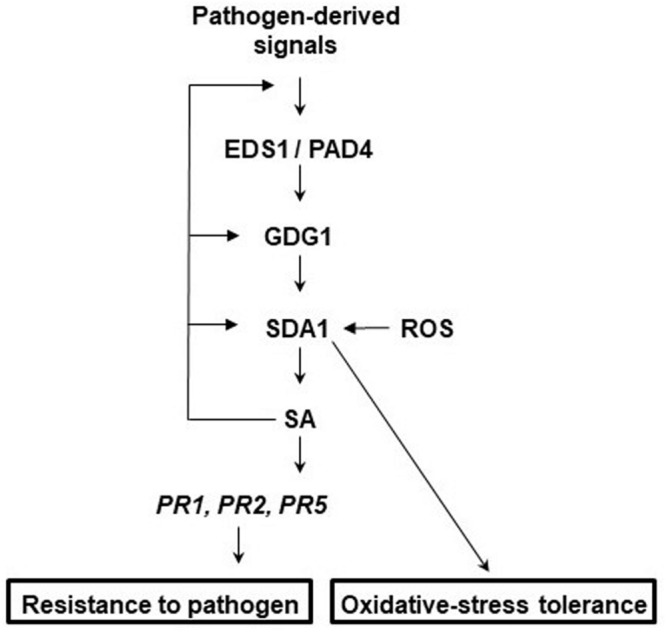
Proposed model for the role of SDA1 in defense signaling and oxidative-stress tolerance. In response to virulent pathogens, expression of *SDA1* is regulated via EDS1, PAD4, and GDG1 (also known as PBS3 and WIN3) but not SID2. SDA1 is at least in part required for pathogen-induced SA biosynthesis, which in turn regulates expression of *PR* genes and defense via NPR1. SA regulates expression of *SDA1* by positive feedback controls, possibly via GDG1. Additionally, control via GDG1 may be dependent on positive feedback to GDG1 at low SA levels and negative feedback at high SA levels. In addition, SDA1 modulates tolerance to oxidative stress.

To determine the epistatic relationship of SDA1 with other defense genes in the SA-mediated defense-signaling pathway, we analyzed expression of *Pst* DC3000-mediated induction of SDA1 in several defense mutants. SDA1 expression was compromised in *ndr1, eds1, pad4, gdg1/pbs3/win3* mutants, and *NahG* expressing plants. However, SDA1 expression was not affected in the *sid2* mutant. This is similar to a previous report that virulent bacterial pathogen-mediated expression of some genes is compromised in the *gdg1* mutant but not in the *sid2* mutant (Wang et al., 2008). Interestingly, both *gdg1* and *sid2* mutants are compromised in *Pst* DC3000-mediated SA accumulation. *SID2* codes for isochorismate synthase, an important SA biosynthetic enzyme (Wildermuth et al., 2001). PBS3/GDG1/WIN3 has been shown to catalyze the conjugation of specific amino acids to SA acyl substrates and has been suggested to allow for coordination of flux through diverse chorismate-derived pathways (Okrent et al., 2009). Our results suggest that SDA1 expression might be modulated by one of these chorismate-derived signals. SA modulates expression of SDA1 by positive feedback regulation, possibly via GDG1/PBS3/WIN3 because SA has been shown to modulate GDG1/PBS3/WIN3 via positive feedback regulation (Jagadeeswaran et al., 2007). Figure 9 shows a proposed model for the role of SDA1 in pathogen defense signaling.

In conclusion, the studies reported here suggest that SDA1 represents a novel class of plant genes required for defense against bacterial pathogens that is part of the SA-mediated defense pathway. Furthermore, SDA1 might be involved in modulating a variety of biotic and abiotic stress responses in addition to response against pathogens. We propose that SDA1 might act to coordinate crosstalk across development, biotic and abiotic stress, ROS and salicylic acid signaling.

## Data Availability Statement

All datasets generated for this study are included in the article/Supplementary Material.

## Author Contributions

AD and RR conceived the original screening and research plans. RR and DK supervised the experiments. AD and PC performed most of the experiments. SC provided technical assistance. AD, PC, PG-B, P-PL, DK, and RR designed the experiments and analyzed the data. AD, PC, and RR wrote the manuscript with contributions of all the authors. RR supervised and completed the writing and agreed to serve as the author responsible for contact and ensures communication.

## Conflict of Interest

The authors declare that the research was conducted in the absence of any commercial or financial relationships that could be construed as a potential conflict of interest.
