# Supplementary material for: Arabidopsis SMALL DEFENSE-ASSOCIATED PROTEIN 1 Modulates Pathogen Defense and Tolerance to Oxidative Stress
Source: Front Plant Sci. 2020 Jun 3;11:703. doi: 10.3389/fpls.2020.00703 (PMC7283558; doi:10.3389/fpls.2020.00703)
Supplement: Supplementary file 1 [file Table_1.pdf]

Supplemental Table S1: DNA oligonucleotide sequences used in the study

Oligonucleotide Name	Sequence (5'-3')	References
FP, forward primer; RP, reverse primer.		
**Establishment of *sda1* mutant alleles**		
Gene-specific-FP (SAIL_390_F08) *sda1-1*	ATGGCGCGCCATGAACGGAACATCGTGGGC	This study
Gene-specific-RP (SAIL_390_F08) *sda1-1*	TAGCATCTGAATTTCATAACCAATCTCGATACAC	This study
Gene-specific-FP (GABI_283D01) *sda1-2*	ATGGCGCGCCATGAACGGAACATCGTGGGC	This study
Gene-specific-RP (GABI_283D01) *sda1-2*	ATATTGACCATCATACTCATTGC	This study
**Cloning of 35S::SDA1-GFP, *SDA1* overexpression and β-estradiol inducible-*SDA1* overexpression**		
GFP/35S/GAL4-FP	ATGAACGGAACATCGTGGGCT	This study
GFP/35S/GAL4-RP	TTGTTTGTGTGTGGTTTTGTGATACTTG	This study
**Cloning of p*SDA1* ::GUS**		
GUS-FP	GTGAATTCTTGACATTGATTTGAGGATAAAGTTTTGAC	This study
GUS-RP	GTCTGCAGTATTGTATATCGTCTTCTCAAAAGTTTTAAAATC	This study
**Primers for site-directed mutagenesis**		
D8A-R	TGG GCT GcC CAG TGG GAC	This study
D8A-F	GTC CCA CTG GgC AGC CCA	This study
Q9R-F	TGG GCT GAC gcG TGG GAC	This study
Q9R-R	GTC CCA Cgc GTC AGC CCA	This study
V27A-F	GGC GCC GcC GTG AGA AGC	This study
V27A-R	GCT TCT CAC GgC GGC GCC	This study
K52A-F	GGG TTA GAC gcA ACC AAA GCT GTA	This study
K52A-R	TAC AGC TTT GGT Tgc GTC TAA CCC	This study
**Primers for RT-qPCR**		
PR1- FP	CATGTGGGTTAGCGAGAAGG	Dutta et al, 2017
PR1-RP	GGCTTCTCGTTCACATAATTCC	Dutta et al, 2017
SDA1-FP	ATG AAC GGA ACA TCG TGG GCT	this study
SDA1-RP	GTCCTTGACCCAACGAAAACC	this study
UBC-FP	TCAAATGGACCGCTCTTATC	Dutta et al, 2017
UBC-RP	CACAGACTGAAGCGTCCAAG	Dutta et al, 2017
