# Supplementary material for: Arabidopsis SMALL DEFENSE-ASSOCIATED PROTEIN 1 Modulates Pathogen Defense and Tolerance to Oxidative Stress
Source: Front Plant Sci. 2020 Jun 3;11:703. doi: 10.3389/fpls.2020.00703 (PMC7283558; doi:10.3389/fpls.2020.00703)
Supplement: Supplementary file 2 [file Data_Sheet_1.pdf]

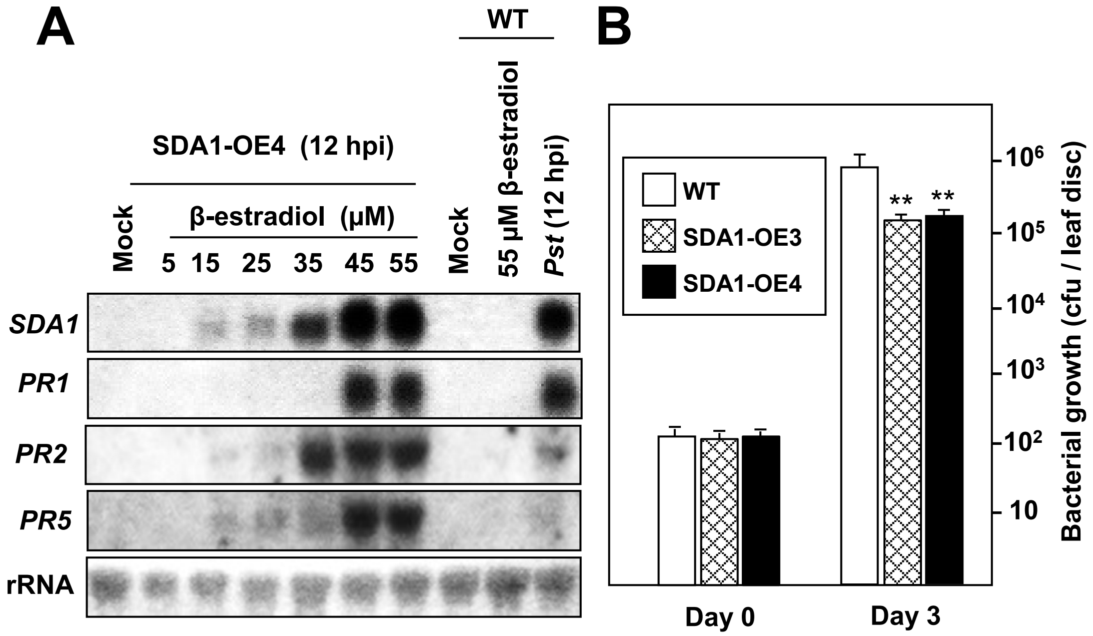

Supplemental Figure S1: Overexpression of the *SDA1* gene induces defense gene activation.

A. Total RNA was isolated from four-week-old SDA1-OE4 (pER8::*SDA1,* line # 4) transgenic plants expressing *SDA1* cDNA from the β-estradiol-inducible system. Rosette leaves were inoculated with indicated concentration of β-estradiol in 0.016% ethanol and tissue was harvested at 12 hpi. WT plants treated with *Pst* DC3000 (*Pst*) were used as a positive control. The blot was stained with methylene blue to show relative amounts of RNA in each lane (28S rRNA), and hybridized with the *SDA1* gene probe, stripped and re-probed with the indicated gene probes.
B. Four-week-old SDA1-OE3 (pER8::*SDA1,* line # 3) and SDA1-OE4 (pER8::*SDA1,* line # 4) plants were treated with 55 μM estrogen and 24 hours later, leaves were inoculated with *Pst* DC3000 at a titer of $5x10^5$ cfu/ml . Eight plants for each genotype were analyzed individually. Data are reported as mean bacterial count (cfu per leaf disc) $\pm$ SD. Statistical analysis was performed by one-way ANOVA followed by Dunnett's test with reference to WT control. Asterisks indicate statistically significant differences (**p<0.01). This experiment was repeated two more times with similar results.

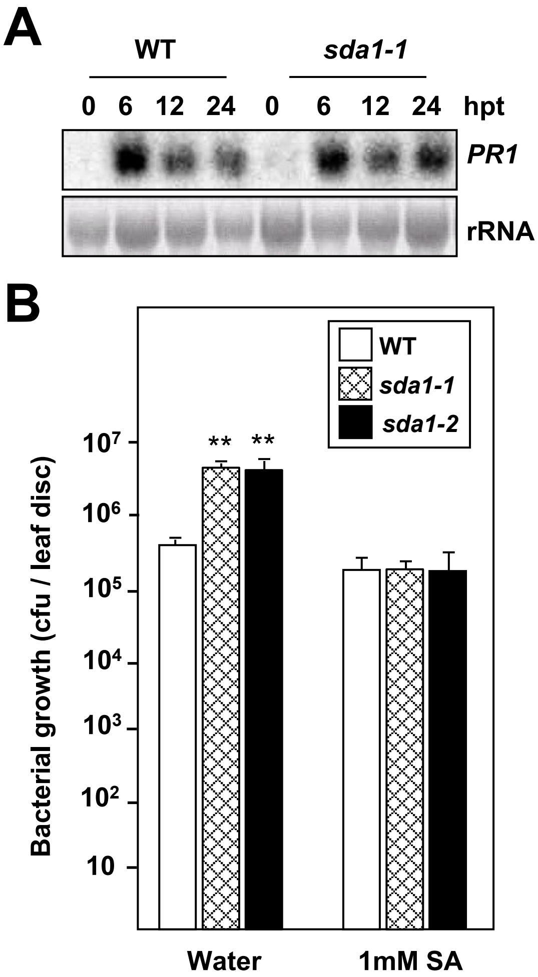

Supplemental Figure S2: SA application rescues *sda1* mutant phenotypes.

A. Plants of the indicated genotypes (four-week-old) were sprayed with 1 mM SA, and tissue samples were harvested at the indicated hours post-treatment (hpt). The blot was stained with methylene blue to determine the relative levels of RNA in each lane (rRNA) and was probed with the *PR1* gene probe.

B. Four-week-old plants of indicated genotypes were sprayed with 1 mM SA or water, and 24 hours later leaves were inoculated with *Pst* DC3000 at a titer of $5x10^5$ cfu/ml in 10 mM $MgSO_4$. Eight plants for each genotype were analyzed individually. Data are reported as mean bacterial count (cfu per leaf disc) $\pm$ SD. Statistical analysis was performed by one-way ANOVA followed by Dunnett's test with reference to WT control. Asterisks indicate statistically significant differences (**p<0.01). This experiment was repeated two more times with similar results.

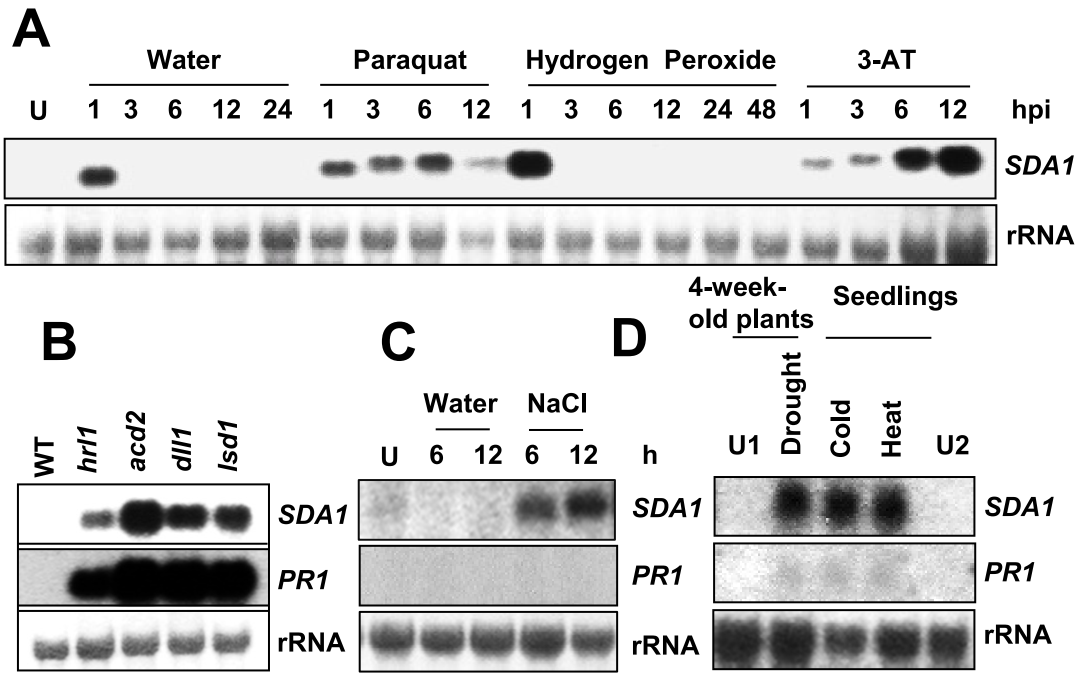

Supplemental Figure S3: Expression analysis of *SDA1* in response to reactive oxygen species (ROS), in lesion mimic mutants, and in response to abiotic stresses.

Northern gel blot analysis was employed to determine transcript levels of *SDA1*. Blots were stained with methylene blue to show the relative amounts of RNA in each lane (rRNA) and were probed with indicated gene probes.

A. Rosette leaves of four-week-old WT plants were infiltrated with 100 µM paraquat, 10 mM hydrogen peroxide, and 0.5 mM 3-AT in water, and tissue samples were harvested at indicated hpi.

B. Lesion positive rosette leaves of four-week-old plants of the indicated genotype and WT plants were harvested and used for RNA isolation.

C. Seven-day-old seedlings were transferred to Whatman paper soaked in water or 200 mM NaCl for indicated time before harvesting for RNA isolation.

D. For drought treatment watering of four-week-old wild type plants was withheld for 7 days prior to harvesting tissue, for cold and heat treatments 7-day-old seedlings were exposed to 4ºC and 37ºC respectively for 48 hours prior to harvesting tissue for RNA isolation. U1 indicates untreated for drought analysis. U2 indicates untreated for temperature analyses.

All experiments were repeated at least twice and similar results were obtained.

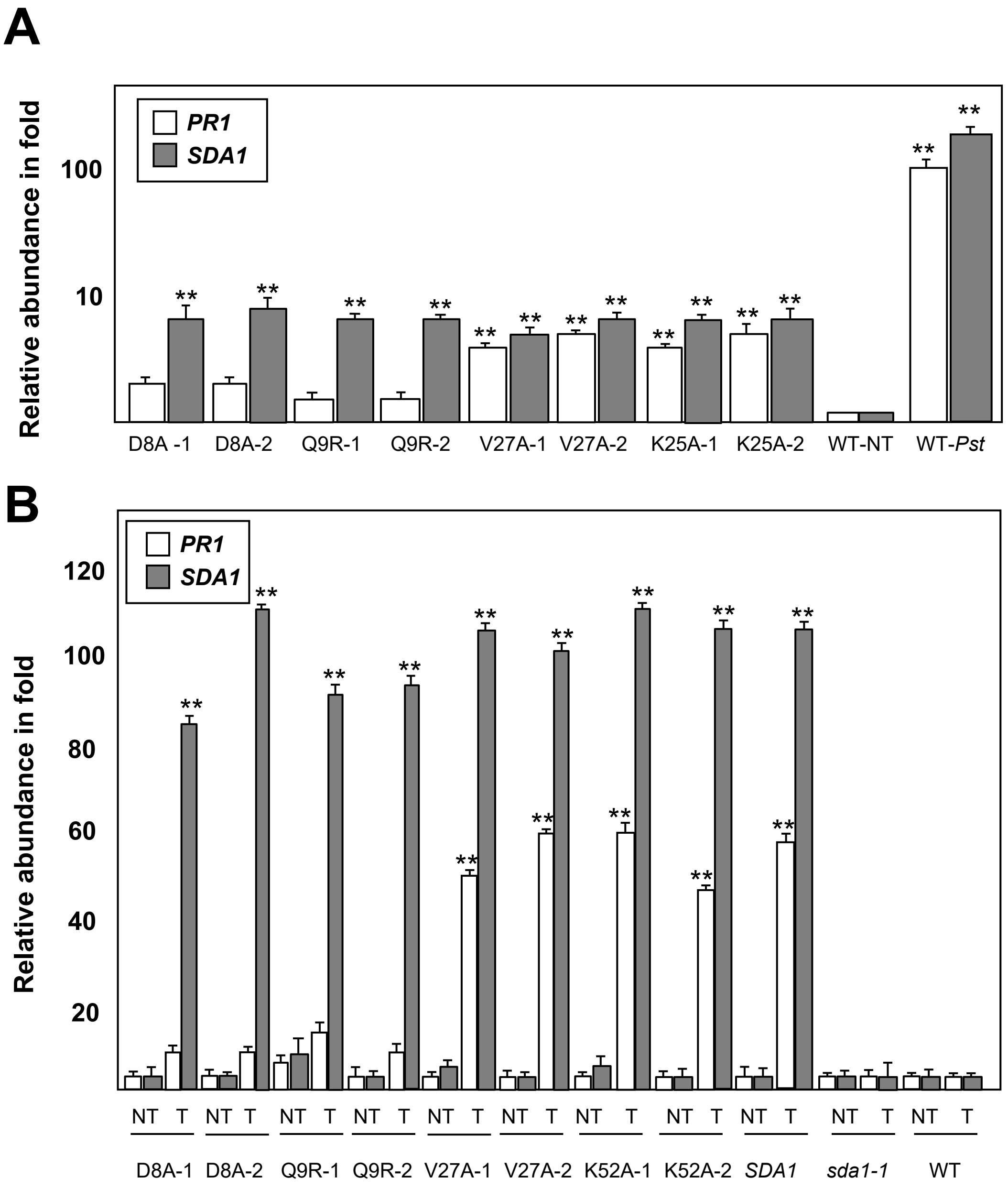
A
PR1
SDA1
Relative abundance in fold
100
10
**
D8A -1
D8A-2
Q9R-1
Q9R-2
V27A-1
V27A-2
K25A-1
K25A-2
WT-NT
WT-Pst
B
PR1
SDA1
Relative abundance in fold
120
100
80
60
40
20
**
NT T
D8A-1
D8A-2
Q9R-1
Q9R-2
V27A-1
V27A-2
K52A-1
K52A-2
SDA1
sda1-1
WT

Supplemental Figure S4: SDA1 D8A and Q9R amino acid mutants are compromised in regulation of *PR1* expression.

A. WT plants expressing indicated point mutants from a 35S promoter. Leaves of four-week-old plants were used for RNA isolation. *PR1* and *SDA1* transcript accumulation was quantified by RT-qPCR. RNA levels were normalized to *UBC* and are shown as the mean ± SEM of three independent biological replicates. Statistical analysis was performed by one-way ANOVA followed by Dunnett's test with reference to WT-NT control. Asterisks indicate statistically significant differences (**p<0.01).

B. *sda1* mutant plants expressing indicated point mutants from an β-estradiol-inducible pER8 promoter. Leaves of four-week-old plants were treated with 55 μM of β-estradiol and tissue samples were harvested from RNA isolation 24 h later. *PR1* and *SDA1* transcript accumulation was quantified by RT-qPCR. RNA levels are normalized to *UBC* and are shown as the mean ± SEM of three independent biological replicates. Statistical analysis was performed by one-way ANOVA followed by Dunnett's test with reference WT-T control. Asterisks indicate statistically significant differences (**p<0.01). SDA1-OE3, *SDA1* expressed from β-estradiol-inducible XVE system (pER8::*SDA1*, line # 3); D8A, Q9R, V27A, K52A are various point mutants of *SDA1* expressed from pER8 promoter.

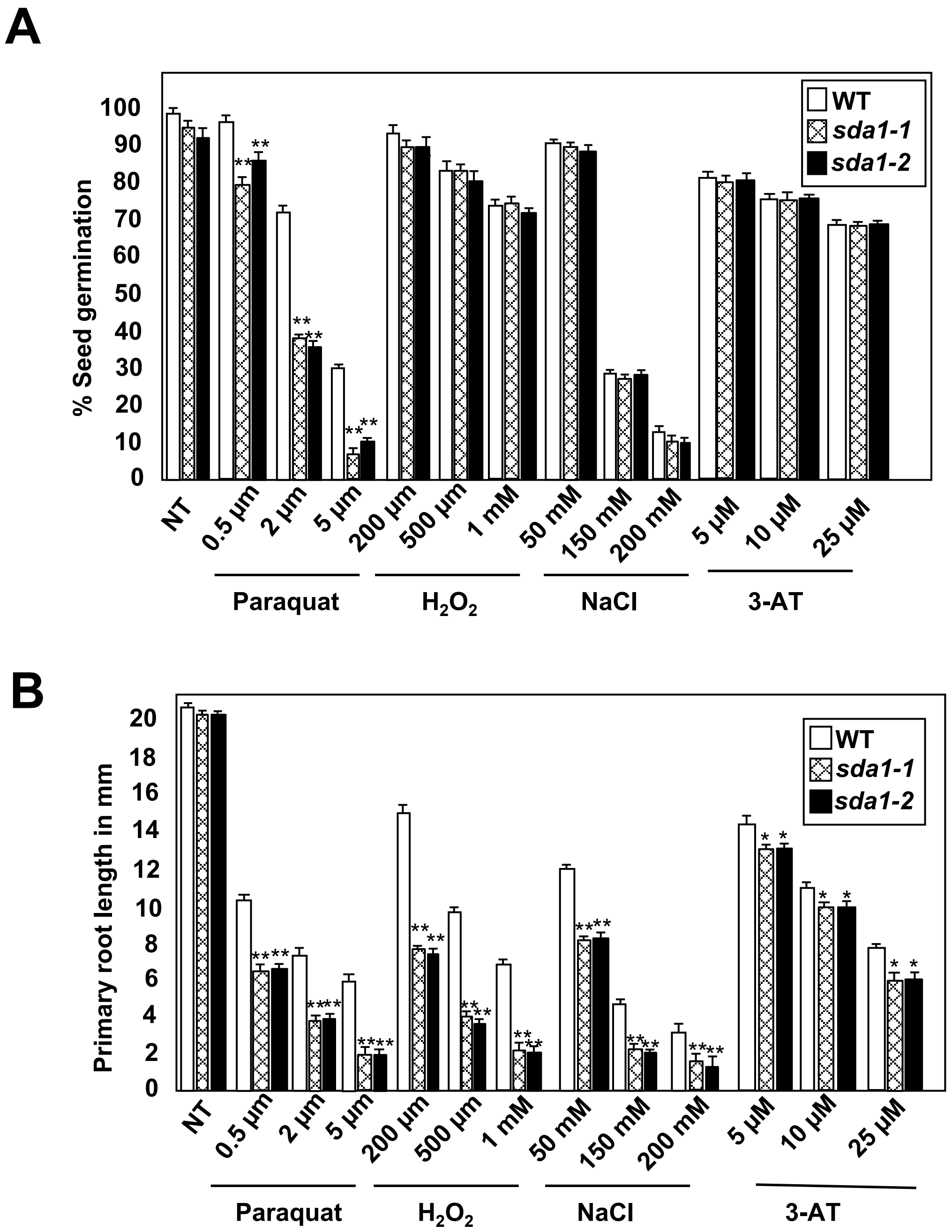

Supplemental Figure S5: SDA1 modulates tolerance to oxidative stress.

A. Percent seed germination of *sda1* mutants and WT in the presence of various oxidative stress inducing chemicals.

B. Root length five-days-old seedlings of *sda1* mutants and WT seedlings in the presence of oxidative stress inducing chemicals. Results are shown as mean root length (n=30; **$p<0.01$, *$p<0.05$). Statistical analysis was performed by one-way ANOVA followed by Dunnett's test with reference to respective WT control of each treatment. Asterisks indicate statistically significant differences. All experiments were repeated twice and similar results were obtained.
